# Supplementary material for: Evaluating Teaching Effectiveness of Medical Humanities in an Integrated Clerkship Program by a Novel Prospective Propensity Score Matching Framework
Source: Int J Environ Res Public Health. 2022 Feb 8;19(3):1882. doi: 10.3390/ijerph19031882 (PMC8834878; doi:10.3390/ijerph19031882)
Supplement: Supplementary file 1 [file ijerph-19-01882-s001.zip › ijerph-1525807-supplementary.pdf]

International Journal of
Environmental Research and Public Health

MDPI

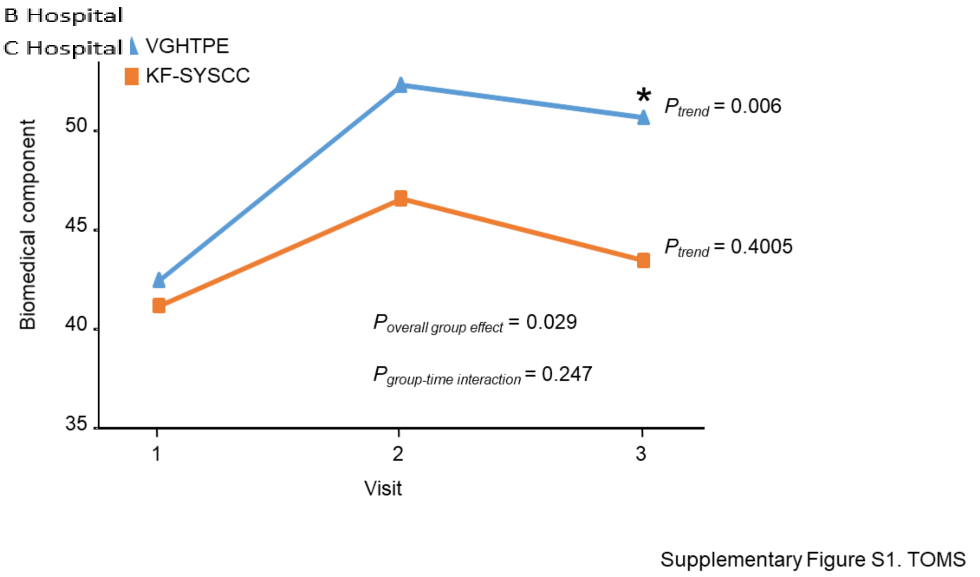

Figure S1. The comparison of TOMS biomedical score between at baseline, at the completion of 3-month clerkship in medicine or surgery, and at the end of the 9-month clerkship program.
